# Redesigning a large school-based clinical trial in response to changes in community practice

**DOI:** 10.1177/1740774511403513

**Published:** 2011-06

**Authors:** Lynn B Gerald, Joe K Gerald, Leslie A McClure, Kathy Harrington, Sue Erwin, William C Bailey

**Affiliations:** a Mel and Enid Zuckerman College of Public Health, and the Arizona Respiratory Center, University of Arizona, Tucson, AZ, USA; b Department of Biostatistics, University of Alabama at Birmingham, Birmingham, AL, USA; c Lung Health Center, University of Alabama at Birmingham, Birmingham, AL, USA

## Abstract

***Background*** Asthma exacerbations are seasonal with the greatest risk in elementary-age students occurring shortly after returning to school following summer break. Recent research suggests that this seasonality in children is primarily related to viral respiratory tract infections. Regular hand washing is the most effective method to prevent the spread of viral respiratory infections; unfortunately, achieving hand washing recommendations in schools is difficult. Therefore, we designed a study to evaluate the effect of hand sanitizer use in elementary schools on exacerbations among children with asthma.

***Purpose*** To describe the process of redesigning the trial in response to changes in the safety profile of the hand sanitizer as well as changes in hand hygiene practice in the schools.

***Methods*** The original trial was a randomized, longitudinal, subject-blinded, placebo-controlled, community-based crossover trial. The primary aim was to evaluate the incremental effectiveness of hand sanitizer use in addition to usual hand hygiene practices to decrease asthma exacerbations in elementary-age children. Three events occurred that required major modifications to the original study protocol: (1) safety concerns arose regarding the hand sanitizer’s active ingredient; (2) no substitute placebo hand sanitizer was available; and (3) community preferences changed regarding hand hygiene practices in the schools.

***Results*** The revised protocol is a randomized, longitudinal, community-based crossover trial. The primary aim is to evaluate the incremental effectiveness of a two-step hand hygiene process (hand hygiene education plus institutionally provided alcohol-based hand sanitizer) versus usual care to decrease asthma exacerbations. Enrollment was completed in May 2009 with 527 students from 30 schools. The intervention began in August 2009 and will continue through May 2011. Study results should be available at the end of 2011.

***Limitations*** The changed design does not allow us to directly measure the effectiveness of hand sanitizer use as a supplement to traditional hand washing practices.

***Conclusions*** The need to balance a rigorous study design with one that is acceptable to the community requires investigators to be actively involved with community collaborators and able to adapt study protocols to fit changing community practices.

## Background

Poor asthma control in children is a well documented public health problem in the US [1,2]. It is associated with frequent exacerbations that cause respiratory symptoms, activity limitation, school absenteeism, and parental work absenteeism [3,4]. Exacerbations also lead to frequent urgent care visits, emergency department visits, and hospitalizations [1,2]. Exacerbations are seasonal with the greatest risk among elementary-age students occurring shortly after returning to school following summer break [5–8].

Outbreaks of viral respiratory infections have been implicated as a cause for this striking seasonal pattern [8–13]. Regular hand washing is the most effective method to prevent the spread of viral respiratory infections [14,15]; unfortunately, achieving effective hand washing practices in schools is difficult [16,17]. Barriers include inadequate time, insufficient soap or paper towels, and inconveniently located sinks [18–20]. Assessment of schools’ physical environments has provided mixed results. A 1998 report of elementary school restrooms in the mid-Atlantic found that 66% of soap dispensers were nonfunctional or insufficiently filled and 33% of automatic hand dryers were inoperable [21]. A 2009 study of primary and secondary school restrooms in New Mexico reported that soap and hand drying were available in 90% of restrooms; however, hand sanitizer was reported in fewer than 2% [22].

To overcome perceived barriers associated with hand washing, some schools have adopted antimicrobial rinse-free hand sanitizers [17,23–25]. Recent studies indicate that hand sanitizers reduce overall infection-related absenteeism among elementary school students by 20–50% [17,18,24–26], respiratory illnesses by 30–50% [17,24,26], and teacher absenteeism by 10% [25]. Use of hand sanitizer in the home has been shown to reduce asthma exacerbations in children and respiratory illnesses among family members [16,27]. Despite these findings, a review by Meadows and Le Saux [28] did not find sufficient evidence to specifically recommend hand sanitizer use in general or among children with asthma; however, a review by Jefferson *et al*. [15] concluded that hand washing did reduce viral respiratory infections, particularly in young children.

To address the questions related to hand washing in general, and the benefit of hand sanitizer in particular, we designed a study to evaluate hand sanitizer use in elementary schools as a mechanism to reduce exacerbations among children with asthma. We describe the study’s design, but more importantly, the process by which revisions were made to the original protocol due to both external and local issues that arose after the original protocol had been funded.

## Methods

### Original study protocol

#### Study design

The original design was a randomized, longitudinal, subject-blinded, placebo-controlled, community-based crossover trial. The primary aim was to evaluate the incremental effectiveness of hand sanitizer use when added to a school’s typical hand washing practices to reduce asthma exacerbations among elementary-age students. To control for school variation and to minimize cross-contamination, individual schools served as the unit of randomization. Crossover between intervention and placebo schools occurred during the summer break between the first and second year. There were two major advantages of the crossover design. The first was the ability to control for the strong seasonal variation in respiratory illnesses that was expected. The second was the ability to maximize school support and participation because all schools would eventually receive the intervention instead of some being relegated to a nonintervention control group.

#### Primary outcome

The primary outcome was the proportion of students who experienced an asthma exacerbation each month defined as one or more of the following: (1) a red (<50% of personal best) or yellow (50–70% of personal best) peak flow meter reading, (2) increased use of quick relief medication from baseline (≥ 4 puffs), or (3) a respiratory-related school absence [29].

#### Setting and recruitment

A single large school district in Birmingham, Alabama that was comprised of 30 elementary schools with approximately 15,000 students was recruited for participation. The system was racially (70% white and 30% black) and economically (33% eligible for free or reduced lunches) diverse. Students with asthma were to be recruited by school nurses who sent information packets home to parents via students. Interested parents were to be encouraged to provide consent to be contacted by study staff or to contact the study staff directly. Prior to obtaining written informed consent from parents and written assent from students, the project was to be explained to both parents and students by the study staff.

#### Study participants

Students who (1) attended one of the participating schools, (2) had physician diagnosed asthma, and (3) could use a peak flow meter were eligible for the study.

#### Intervention

The intervention consisted of using a nonalcohol-based hand sanitizer to supplement the schools’ typical hand washing practices. During the first year, all schools were to receive hand sanitizer containing either an active ingredient or placebo that was dispensed from permanently mounted or free standing 1.8L dispensers (3600 applications) that provided amounts appropriate for elementary-age students. All hand sanitizer was to be provided by the study and was to be placed in the lunchrooms and classrooms. No soap, paper towels, or air dryers were to be provided to the schools.

Students, faculty, and staff in all schools would be instructed on hand washing and hand sanitizer techniques based on the ‘Always Be Clean’ (ABC) hand hygiene program developed by Woodward Laboratories Incorporated. Hand washing with soap and water was to be promoted after using the restroom and when visible dirt was on the hands as recommended by the CDC [30]. Hand sanitizer use was to be promoted as a supplement to hand washing upon arrival at the classroom, before lunch, after using the restroom, at the end of the day, and after sneezing or coughing as recommended by ABC. Grade-level appropriate instruction was to be provided at the beginning of each school year with reinforcement on the first day of each month. Faculty and staff were to be provided continuing education prior to the start of each school year.

#### Hand sanitizer active ingredient

A nonalcohol-based hand sanitizer containing benzalkonium chloride (0.13%) was chosen for the study. Benzalkonium chloride, was designated ‘GRAS’ (generally regarded as safe) for topical antiseptic applications by the Food and Drug Administration (FDA) [31]. It was effective against Gram-negative and Gram-positive bacteria associated with nosocomial infections and many viruses associated with upper respiratory infections including human coronavirus and adenovirus [32]. It also had been used previously in several large school-based studies without adverse events [17,26,33,34]. Another important consideration was the availability of a placebo without antimicrobial effects [17].

Both alcohol-based and nonalcohol-based hand sanitizers were initially considered, but community concerns regarding alcohol-based products and their flammable nature [14,35] and the potential for misuse made them a less desirable choice [36]. There were also preliminary data to suggest that alcohol-based products might be inferior to nonalcohol-based products [33,34]. For example, the antimicrobial activity of benzalkonium chloride had been shown to increase over multiple, consecutive washes whereas the antimicrobial activity of ethyl alcohol tended to decrease over time [33,34]. Both had better degerming activity than soap and water [33,34], but 50% of those using ethyl alcohol reported hand pain or discomfort whereas no one using benzalkonium chloride reported similar symptoms [33].

#### Data collection

A web-based monitoring system (Asthma Agents) developed in collaboration with Blue Cross and Blue Shield of Alabama was to be used to collect daily data without overburdening the schools or interrupting learning activities [29]. Peak flow meter (PFM) readings and school absences were to be recorded daily by students and verified by teachers and/or school nurses. Quick relief medication (Proventil® HFA) for in-school use was to be provided at no charge to all children enrolled in the study by Merck and Company, Incorporated. A Doser™ was to be attached to each student’s inhaler to record each actuation of quick relief medication automatically. The count was to be recorded every 2 weeks by the study staff.

Compliance with hand hygiene recommendations was to be estimated based on the frequency of refills of hand sanitizer in each classroom and in the lunchroom. Refills were to be stored in the housekeeping office at each school and the custodial staff recorded refill dates. This method was chosen to minimize the burden on classroom faculty and staff. Since all hand sanitizer was to be supplied by the study, the total amount provided to each school was to be monitored.

Other explanatory variables and secondary outcomes including the student’s age, gender, race, asthma severity, quality of life, asthma control, and household smoking exposure were to be collected during bi-annual phone interviews with parents.

#### Data analysis

Data from the Asthma Agents system and the Doser™ were to be used to calculate the proportion of students in each group who experienced an exacerbation. Generalized estimating equations were to be used to model the marginal rate of exacerbations, defined as the proportion of students within each school who experienced at least one asthma exacerbation each month, while controlling for correlation between observations within each student and between students within each school. Adjustment for individual level factors such as the student’s age, race, gender, and asthma severity were to be undertaken also. The study was powered to detect a time averaged difference of 7.5–10% between the exacerbation rates of the intervention and control schools given a sample size between 468 and 650 students with asthma.

#### Safety monitoring

The study was approved and to be monitored by the Institutional Review Boards at the University of Alabama at Birmingham and the University of Arizona. A Data Safety and Monitoring Board (DSMB) was established to monitor adverse events. Two asthma safety events were mandated as reportable: (1) a red PFM reading (<50% of personal best) with symptoms 3 days in a row and (2) use of more than 30 puffs of quick relief medication in a 2 week period for those whose medication was kept in the school office or more than 40 puffs in a 2 week period for students who self-carried. Self-carry of quick relief medication was allowed for any student who had the maturity to use it appropriately provided that the student’s physician completed an authorization form. Parents, teachers and school staff were to be provided handouts describing potential side effects of hand sanitizer use (i.e., flaky skin, lesions, rash, etc.) and how to report them.

### Major events and protocol revision overview

After receipt of the grant award in August 2007 (See Table 1 for Study Timeline), three events occurred that required major modifications to the original study protocol: (1) concerns arose regarding the safety of benzalkonium chloride; (2) no nonalcohol-based hand sanitizer substitute was available; and (3) community standards changed regarding typical hand hygiene practices in the schools.

**Table 1:** Study timeline

Date	School year	Activity
October 2006	2006–2007	Submission
August 2007	2007–2008	Funding Award
January–May 2008	2007–2008	School Recruitment
March 2008–May 2009	2007–2008	Parent/Student Recruitment
	2008–2009	
August 2009	2009–2010	Intervention Starts
August 2010	2010–2011	Crossover

While responding to an IRB requirement to re-review product safety data following grant awards, we found new data indicating that benzalkonium chloride: (1) induced moderate genotoxic effects in eukaryotic cells [37], (2) produced histological changes (hyperplasia, incomplete keratinization, loss of the granular layer, acantholysis, and necrosis) in organ-cultured skin [38], (3) induced biofilm formation (a matrix of cells attaching to each other and a surface) of some bacterial species [39], and (4) possibly caused allergic contact dermatitis [40]. These concerns prompted a search for another nonalcohol-based product but no substitute could be found. The only viable option was to switch to an alcohol-based product. While this alleviated many of the safety concerns related to benzalkonium chloride, it prevented the use of a subject-blinded design because there was no placebo for an alcohol-based product.

Between the time of the grant development and its award, the schools’ typical hand hygiene practices changed substantially due in large part to changes in the community’s perceptions regarding disease risk. School principals, faculty, and parents had become convinced that hand sanitizer was necessary for good hand hygiene within the schools. There was considerable fear that hand washing alone was not sufficient to prevent ‘the spread of germs’. This perception was reinforced by a methicillin-resistant *Staphylococcus aureus* (MRSA) outbreak in several schools prior to the study. Because of these concerns, hand sanitizer use increased dramatically such that many schools required parents to purchase personal hand sanitizer as a supply item. Despite reassurance by the local health department that hand washing remained the best option for hand hygiene, community perceptions were so strong that a hand washing only arm was no longer a viable option.

### Revision process

The decision to change a study protocol after a grant award is a difficult one that involves many important stakeholders. For this trial, important stakeholders included the National Heart, Lung, and Blood Institute (NHLBI) and its designee (project officer), our Data Safety and Monitoring Board (DSMB), our Institutional Review Board, and the community as represented by school administration, faculty and staff, and parents. Our goal was to maintain the scientific integrity of the study while adapting to safety concerns and changes in the community’s preferences.

Chronologically, the first major decision involved the change from the original hand sanitizer with its corresponding placebo to an alcohol-based product without one. While the safety concerns that prompted this decision were mostly theoretical, we agreed that ensuring participant safety, especially among children, warranted erring on the side of caution. The principal investigator and key study personnel made this decision in close collaboration with the NHLBI project officer and DSMB over the course of multiple teleconferences. The primary goal was to ensure that the redesigned study addressed an important research question using a methodologically rigorous design without compromising student safety. The school system administration (superintendent and chief nursing officer) were appraised of the rationale for the proposed changes and were asked to approve them prior to implementation.

Because there was no placebo for the alcohol-based product, it was no longer possible to conduct a subject-blinded design for a direct test of the effectiveness of hand sanitizer as a supplement to hand washing. Instead, we chose to compare the effectiveness of a standardized two-step hand hygiene process (hand washing plus hand sanitizer) with handwashing only. Schools would be randomly assigned to use study-provided hand sanitizer and hand soap (intervention arm) or to hand washing only (usual care arm). Crossover would still occur after the first year.

When we met with school principals and parent advisory groups to discuss the revisions, including randomization of some schools to hand washing only, we learned that personal hand sanitizer use had become ubiquitous in many schools. We asked schools to discourage personal hand sanitizer use (e.g., removing hand sanitizer from their required supply lists) by arguing that hand washing was the preferred method of hand hygiene. The local health department reinforced this message on our behalf, but parents and school faculty countered that personal hand sanitizer use was surmounting existing barriers to adequate hand washing and that they were reticent to restrict its use. They also pointed out the difficulty of enforcing restrictions against personal hand sanitizer use while at school.

Unfortunately, we were unable to secure any restrictions which meant that instead of the usual care arm being a hand washing only arm, it would now also include a variable amount of personal hand sanitizer use as well. The unexpected increase in personal hand sanitizer use presented a potential bias to the null effect; to control for this possibility we developed instruments to monitor the schools’ hand hygiene practices more carefully.

All of the revisions occurred prior to participant enrollment; therefore, no changes to the consent process were necessary.

### Revised study protocol

#### Revised study design

The revised protocol was a randomized, longitudinal, community-based crossover trial that compared the effectiveness of a standardized two-step hand hygiene process (hand hygiene education plus study-provided hand sanitizer and hand soap) with that of usual care (a variety of school-specific hand hygiene practices).

#### Revised primary outcome

No change.

#### Revised setting and recruitment

No change.

#### Revised study participants

No change.

#### Revised intervention

The revised intervention consisted of a two-step hand hygiene practice that included regular hand washing with soap and water supplemented by hand sanitizer use. To overcome potential resource barriers to adequate hand hygiene practices in intervention schools, the study provided intervention schools with alcohol-based hand sanitizer, hand soap, and needed refills. Hand soap dispensers were installed by study personnel and hand sanitizer was made available in disposable bottles in all intervention restrooms, health rooms, and classrooms with a sink.

To our surprise the installation of dispensers became an important issue. Originally, we used the installation of permanent hand sanitizer dispensers as an incentive for schools to participate. Since the hand sanitizer dispensers would hold product with either an active ingredient or placebo, installing them at the beginning of the study in all schools did not pose a problem following crossover. After the redesign, the lack of a placebo created the potential for a carry-over effect if Year 1 intervention schools were to continue using the dispensers during Year 2 when assigned to usual care. We proposed eliminating permanent dispensers altogether, but discontinuation became an issue with the schools as they wanted us to honor our original commitment.

A compromise was reached by installing hand soap dispensers, instead of hand sanitizer dispensers, in each school prior to the start of their intervention year. Schools were concerned that removing dispensers after the intervention year was over might damage walls so we agreed to leave them in place but we would no longer supply refills. We deemed that the potential use of the hand soap dispensers during the usual care year posed less of a carry-over/contamination risk than the use of hand sanitizer dispensers would.

In the revised design, the ‘Always Be Clean’ hand hygiene curriculum was replaced with the Centers for Disease Control and Prevention’s (CDC) School Network for Absenteeism Prevention (SNAP) program (http://www.itsasnap.org/index.asp) because the ABC program was a branded program of Woodward Laboratories Incorporated, the company which produces the benzalkonium chloride hand sanitizer. Hand washing with soap and water was promoted after using the restroom and when visible dirt was on the hands as recommended by the CDC [30]. Hand sanitizer use was promoted as a supplement to hand washing upon arrival at the classroom, before lunch, after using the restroom, and after sneezing or coughing.

Only schools in the intervention arm received the annual training and monthly refreshers; usual care schools did not receive any hand hygiene education or any hand hygiene supplies. They were expected to maintain their customary hand hygiene practices. These practices varied across schools and included hand washing and personal hand sanitizer use of differing quality and frequency. Some of this variation was attributable to the inconsistent supply of hand hygiene resources among some schools. Because of this variability, usual care schools might not be able to maintain the hand hygiene practices necessary to minimize the spread of respiratory viruses among the student population.

#### Revised hand sanitizer/active ingredient

Ethyl alcohol (62% solution) in foam (Purell® instant hand sanitizer foam)*.*

#### Revised data collection

The data collection procedures for the primary outcome remained unchanged; however, two new data collection instruments were designed to evaluate the school environment as it related to hand hygiene practices. The first instrument was used to conduct a one-time baseline assessment of each school’s ‘fixed’ environmental facilities with regard to the availability, type and working condition of sinks, soap dispensers, hand sanitizer dispensers and hand drying devices (i.e., paper towels or air-dryers). The availability of hot and cold water and type of faucet mechanisms available (e.g., spring-loaded, automatic, manual-turn) was also recorded.

The second instrument was used for monthly monitoring of the availability of hand hygiene resources at each school. The type of hand hygiene supplies available at each school (usual care and intervention) and their availability, that is, liquid or bar soap, hand drying mechanism, hand sanitizer, and hand hygiene instructions are recorded. General cleanliness and structural condition also were noted. Data were used to describe hand hygiene resource availability in the usual care schools and to monitor implementation of the two-step hand hygiene program in the intervention schools.

Since hand sanitizer was recommended as a supplement to standard hand washing in a setting of limited school resources, it was deemed important to estimate the incremental value of hand sanitizer use. Therefore, an economic analysis was added as a study aim to estimate the additional costs of hand sanitizer use. A careful cost accounting of the usual care and intervention activities is being conducted. Cost and effectiveness data will be combined to conduct a cost-effectiveness analysis comparing the two-step hand hygiene intervention with usual care using dollars per averted asthma exacerbation as the primary outcome.

#### Revised data analysis

Power calculations were revised based on the updated study design using the monthly frequency of episodes of poor asthma control (EPAC) as the primary outcome (Table 2). Two general scenarios were assumed: one where there was no carryover effects (data from both years) and one where there were such effects (each year evaluated separately). GEE was used to account for the correlation within a child over time as well as the correlation among children within the same school.

**Table 2:** Revised power calculations

No carryover effect (two school years compared)			Carryover effect present (each school year compared)		
Number of Children Per School (30 schools)	Decrease in frequency of EPACs, due to intervention (%)	Power (%)	Number of Children Per School (30 schools)	Decrease in frequency of EPACs, due to intervention (%)	Power (%)
14	7.5	74	14	7.5	48
	10	92		10	71
15	7.5	77	15	7.5	50
	10	93		10	71
16	7.5	79	16	7.5	54
	10	94		10	74

EPAC = Episodes of Poor Asthma Control.

Carryover (or residual) effects occur when the effect of an intervention during the first time period persists into the second time period. The persistence of the effect may bias the measured effect of the second intervention. It is possible that educational intervention could lead to sustained changes in hand hygiene practices at interventions schools that might persist into the second year; however, the presence of a summer break provides a 10-week ‘wash out’ period that should minimize any carryover effect since no reinforcement is provided to original intervention schools during the second year. Research has shown that changes in behavioral practices typically decline by 40–80% after 6 weeks without reinforcement [41]. A recent review of hand hygiene interventions in health care workers noted that without reinforcement the changes in hand hygiene behaviors were sustained for less than 1 week [42]. A 1 month baseline period at the beginning of each school year was planned to measure potential carryover effects.

#### Revised safety monitoring

No change.

## Trial status

Enrollment was completed in May 2009 with a total of 527 students enrolled in 30 schools (mean = 17.6 per school). The enrollment per school exceeded the estimate used to calculate the study’s power. The intervention began in August 2009 and will end in May 2011.

## Conclusions

One of the most difficult aspects of the academic-community partnership is the lag time between study development, funding, and implementation. During this interval, key stakeholders, perceptions, and standard practices within the community can change, often in an unpredictable manner which is outside the control of the research team. At the same time, new scientific discoveries may emerge that render the original study assumptions obsolete. Because of this dynamic environment, the community-based researcher must be responsive to changes that may undermine the original study design or collaborations with community partners.

We have found that a study team with a broad range of skills, talents, and interests is well positioned to respond to a quickly changing environment. Not all team members are equally suited to serve as liaisons with community partners. In school-based research, team members with prior work experience in the schools (e.g., former administrators, teachers, or coaches) often can establish more effective collaborations with school personnel than ‘academicians’. These team members can use their previous relationships to identify and gain access to the key stakeholders within the school system. Additionally, they can navigate a cultural work environment that is often quite different from the academic setting. Having team members with this background is critically important for school-based research.

In retrospect, the most crucial decision that impacted our original research design was the decision to abandon the nonalcohol-based hand sanitizer containing benzalkonium chloride. Without this product and its corresponding placebo, we were unable to conduct a subject-blinded design. Initially, we felt confident that the product was safe and effective; however, the *in vitro* data that subsequently emerged cast doubts on the product’s previously established safety record. The new data emerged at a point in the study cycle where little time was available to contemplate the decision without substantially delaying implementation. We also were sensitive to safety concerns given that we were working with a vulnerable population (children) in a challenging research setting (schools). We believed that a smaller body of *in vitro* findings questioning the product's safety trumped a larger body of *in vivo* findings demonstrating safety that were obtained through industry-funded, manufacturer-led research.

The impact of eliminating the subject-blinded design was magnified by the abrupt change in community hand hygiene practices within the schools. If personal hand sanitizer use had not been prevalent in usual care schools, the crossover design would have been a robust test of hand sanitizer as compared with hand washing alone. However, the ubiquitous nature of personal hand sanitizer use forced us to rely on what had been reported previously to be substandard hand hygiene practices within the schools due to significant structural barriers. By supplying the education and resources to ensure that the barriers to effective hand hygiene in the intervention schools were eliminated, we were able to evaluate the best possible hand hygiene practice (hand washing plus hand sanitizer use) against a variety of usual care practices. Unfortunately, the study no longer can measure the effectiveness of hand sanitizer use directly as a supplement to traditional hand washing practices. If the trial results are negative, the possibility of bias created by personal hand sanitizer within usual care schools may be the primary contributing factor.

Community-based clinical trials are needed to establish the effectiveness of interventions under real-world conditions [43]. A methodologically rigorous study design is important to achieve this goal, but the community-based researcher must also respect community beliefs and practices [44]. Failure to address them satisfactorily may compromise the ethical conduct of research, particularly in vulnerable populations. It also may be counterproductive as the community partners are vital to the translation of research knowledge into sustainable practices.
